# Resveratrol Promotes Autophagy to Improve neuronal Injury in Parkinson’s Disease by Regulating SNHG1/miR-128-3p/SNCA Axis

**DOI:** 10.3390/brainsci13081124

**Published:** 2023-07-25

**Authors:** Dong-Fang Shen, Hui-Ping Qi, Wei-Na Zhang, Wen-Xu Sang

**Affiliations:** Department of Neurology, The Fourth Affiliated Hospital of Harbin Medical University, No.37, Nangang District, Harbin 150001, China; qihuiping2005@163.com (H.-P.Q.); weina850425@163.com (W.-N.Z.); 15004660711@163.com (W.-X.S.)

**Keywords:** resveratrol, SNHG1, miR-128-3p, SNCA, Parkinson’s disease

## Abstract

Background: Parkinson’s disease (PD) is seriously threatening the health and life quality of the elderly, who have a high incidence and high disability rate. Resveratrol (RES) was reported to play a protective role in PD. However, the functions and potential mechanism of RES in PD remain unclear, which need to be further explored. Methods: Human neuroblastoma cells (SH-SY5Y and SK-N-SH) were subjected to 1-Methyl-4-phenylpyridium (MPP+) induction to construct a cell model of PD. Cell viability was evaluated using CCK-8. The gene expression was evaluated using qRT-PCR and western blot. Luciferase activity assay and RIP were performed to validate interactions among SNHG1, miR-128-3p and SNCA. Results: Our results exhibited that RES reduced SNHG1 and SNCA expression but elevated miR-128-3p expression in human neuroblastoma cells upon MPP+ induction. Functionally, RES resulted in the promotion of cell autophagy in MPP+-induced human neuroblastoma cells, while these influences were abolished by SNHG1 overexpression. Mechanistically, SNHG1 could indirectly elevate SNCA expression via sponging miR-128-3p. Moreover, SNCA overexpression reversed SNHG1 silencing-induced cell autophagy in MPP+-induced human neuroblastoma cells upon RES pre-incubation. Conclusions: RES prevented MPP+-induced repression of cell autophagy through inhibiting the SNHG1/miR-128-3p/SNCA axis, suggesting that RES might play a preventive effect on PD progression.

## 1. Background

Parkinson’s disease (PD) is a common neurodegenerative disease in the elderly. The primary pathogenesis is the reduction of the synthesis of dopamine and the increase of acetylcholine in the substantia nigra cells located in the midbrain, thereby resulting in motor retardation, uncontrollable resting tremor, muscle rigidity, motor retardation and autonomic nerve dysfunction [1]. In the global disease burden study published in 2016, the number of PD patients account for about 1–5% of the population over 60 years old; approximately 6.1 million people around the world have been diagnosed with PD, which seriously lowers the quality of life of PD patients and brings a great economic burden to PD patients [2]. Currently, dopamine replacement (Levodopa) is the most effective and widely recognized method, but as the symptoms of PD continue to progress, the effectiveness of dopamine replacement is weakened and adverse side effects, such as movement disorders, appear [3]. Therefore, it is urgent to deeply explore the pathogenesis behind PD and effective preventive drugs for PD.

Autophagy is a process of lysosomal degradation of cytoplasmic proteins and organelles, which is highly conserved in evolution and exists widely in various animals, including human beings [4]. At present, a growing number of studies have demonstrated the process of cell autophagy is closely related with multi-diseases [5]. Indeed, there was substantial evidence that cell autophagy was considered to be implicated in the progression of PD [6]. For example, Yan Su et al. suggested that MPP^+^-induced inhibition of the autophagy process in SH-SY5Y and PC12 cells and plumbagin could promote autophagy to alleviate cell injury caused by MPP^+^ induction [7]. In addition, a study published in 2021 suggested that promoting the autophagy process may be a preventive direction for PD [8]. Here, we will investigate the molecular regulatory mechanism behind PD from the direction of autophagy.

Resveratrol (RES), chemically known as 3,4’,5-trihydroxytrans stilbene, is a natural plant antitoxin, a substance produced by plants to protect them from environmental stress and pathogen invasion [9]. RES could be extracted from Veratrum album (white hellebore) and Polygonum cuspidatum (Japanese Knotweed) [9]. At present, RES has been widely investigated in various diseases, such as cancers, nervous system diseases and has exhibited powerful capabilities against multi-diseases [10,11]. Furthermore, in PD, RES was reported to promote autophagy, thereby achieving neuroprotective effects in PD [12]. However, the molecular regulatory mechanism of RES-mediating autophagy in PD is still unclear. Therefore, in this study, we focused on this point. 

The long non-coding RNAs (lncRNAs) are classified as non-coding RNAs which ubiquitously exist in eukaryotes and take on regulatory roles in biological processes [13]. Accumulating evidence has elucidated that lncRNAs were implicated with PD progression through regulating the autophagy process [14]. For example, lncRNA OIP5-AS1 prompted cell autophagy of MPP^+^-induced SH-SY5Y cells to attenuate α-synuclein aggregation and toxicity through mediating the miR-126/PLK2 axis [14]. It has been reported that lncRNA small nucleolar RNA host gene 1 (SNHG1) expression was elevated in PD models in vitro and in vivo and SNHG1 silencing could relieve the nerve injury of PD through stimulating autophagy and suppressing cell apoptosis [15]. Another study suggested that RES could result in downregulation of SNHG1 expression to reduce amyloid β-protein (Aβ) in Alzheimer’s disease [16]. Hence, we rationally speculate that the downstream molecule of RES might be involved in SNHG1 to regulate cell autophagy in PD.

Similarly, miRNAs are another member of non-coding RNAs in eukaryotes and their importance in biological processes have been illustrated by numerous studies. Prior research suggested the miR-128-3p level abnormally declined in a 1-methyl-4-phenyl-1,2,3,6-tetrahydropyridine (MPTP)-established animal model of PD, and miR-128-3p overexpression could attenuate the MPTP-promoted apoptosis of hippocampal neurons, indicating that miR-128-3p was a beneficial regulator in PD [17] and also suggesting miR-128-3p might be equipped with crucial functions in PD. However, there is lack of research about the mechanism of miR-128-3p in PD, which deserve to be further investigated. 

The alpha-synuclein (SNCA) gene can encode α-synuclein protein, which plays a core role in the pathogenesis of PD. The α-synuclein protein can cause toxicity and neuronal death, thus leading to the onset and development of PD [18]. Previous research has extensively reported that SNCA was closely related with cell autophagy [19]. For instance, Piperine could promote cell autophagy in a SNCA/α-synuclein-induced PD model (in vivo and in vitro) to mediate neuroprotective effects in PD through accelerating the degradation of SNCA [19]. This above-mentioned evidence expounded the crucial functions of SNCA in PD on cell autophagy.

It is well accepted that lncRNAs sponge miRNAs to charge expressions of downstream targeted genes and further modulate multi-biological processes [20]. Combined with bioinformatics analysis, we found that SNHG1 and SNCA possess potential binding sequences on miR-128-3p. Considering the previous studies, we put forward the hypothesis that RES promotes autophagy by regulating the SNHG1/miR-128-3p/SNCA axis, preventing neuronal injury in PD, which suggests that RES might play a preventive effect on PD progression and the SNHG1/miR-128-3p/SNCA axis might be a novel axis for PD treatment.

## 2. Methods

### 2.1. Cell Culture and Treatments 

Human neuroblastoma cells including SH-SY5Y and SK-N-SH cells (ATCC, Manassas, VA, USA) were maintained in Dulbecco’s Modified Eagle’s medium (DMEM) medium (Thermo Fisher Scientific, Waltham, MA, USA) containing 10% fetal bovine serum (FBS) (Gibco, Carlsbad, CA, USA), 100 U/mL penicillin and 0.1 mg/mL streptomycin under condition of 5% CO_2_ airflow at 37 °C.

Cells were subjected to 1 mM MPP^+^ induction for 24 h to construct the cell model of PD. Different concentrations of RES (25, 50 and 100 μM) were applied to pre-treat SH-SY5Y and SK-N-SH cells for 12 h before MPP^+^ induction. A total of 10 mM 3-Methyladenine (3-MA) was applied to treat SH-SY5Y and SK-N-SH cells for 24 h to block the process of cell autophagy.

### 2.2. Cell Transfection

The small interfering RNA against SNHG1 (sh-SNHG1), overexpression vector of SNHG1 (oe-SNHG1), miR-128-3p mimic/inhibitor and their negative control (sh-NC, oe-NC, NC mimic, NC inhibitor) were acquired from GenePharma (Shanghai, China). SH-SY5Y and SK-N-SH cells were transfected with aforementioned substances using Lipofectamine™ 3000 (Invitrogen, CA, USA) following the manufacturer’s instruction. 

### 2.3. Cell Counting Kit-8 (CCK-8) Assay

The cell viability was evaluated using a CCK-8 assay. Firstly, human neuroblastoma cells were planted onto 96-well plates for 48 h. After transfection and/or treatments, 10 µL CCK-8 solution was supplied to each well and incubated for 2 h. A microplate reader (Thermo Fisher Scientific) was employed to measure the absorbance at 450 nm.

### 2.4. Western Blot 

The proteins were extracted from SH-SY5Y and SK-N-SH cells using Radioimmunoprecipitation(RIPA) lysis buffer (Beyotime, Biotechnology, Shanghai, China). A Bicinchoninic acid (BCA) Assay Kit (Beyotime Biotechnology) was used to measure the concentration of total protein and proteins were separated by SDS-PAGE. Afterwards, the separated proteins in sodium dodecyl sulfate-polyacrylamide gel electrophoresis (SDS-PAGE) were transferred onto a polyvinylidene fluoride (PVDF) membrane. After blocking with skimmed milk (5%) for 1 h, primary antibodies p62 (1:1000, #ab240635, abcam), Beclin-1 (1:2000, ab207612, abcam), LC3B (1:2000, #ab192890, abcam) and β-actin (1:1000, #ab8226, abcam) were performed to incubate the PVDF membranes overnight at 4 °C. Subsequently, secondary antibodies were used to incubate the membranes for 1 h, and an ECL kit (Thermo Fisher Scientific) was used to visualize the protein bands. Image J software (NIH-Image, Bethesda, MD, USA) was applied to analyze the gray values.

### 2.5. Quantitative Real Time Polymerase Chain Reaction (qRT-PCR)

TRIzol reagent (Invitrogen, CA, USA) was used to extract RNAs from SH-SY5Y and SK-N-SH cells. A Prime Script Reverse Transcription Reagent Kit (TaKaRa, Dalian, China) and a first-strand cDNA synthesis kit (Sangon, Shanghai, China) were applied to synthesize cDNA. SYBR Premix Ex Taq II Kit (Takara) was employed to conduct the qPCR process. All data were calculated by using the 2^−ΔΔCt^ method. β-actin was used as the control.

The primer sequences were presented as follows (5′-3′):

SNHG1 (F): ACGTTGGAACCGAAGAGAGC
  SNHG1 (R): GCAGCTGAATTCCCCAGGAT
  miR-128-3p (F): GTCGTATCCAGTGCAGGGTCCGAGGTATTCGCACTGGATAC
  GACAAAGAG
  miR-128-3p (R): CGGTCACAGTGAACCGGT
  SNCA (F): TGTAGGCTCCAAAACCAAGG
  SNCA (R): TGTCAGGATCCACAGGCATA
  β-actin (F): TGGCACCACACCTTCTACAA
  β-actin (R): CCAGAGGCGTACAGGGATAG

### 2.6. Dual Luciferase Activity Assay 

To validate the interactions among SNHG1, miR-128-3p and SNCA, we conducted a dual luciferase activity assay. Shortly, the wild-type (WT) or mutant-type (MUT) sequences of SNHG1 or SNCA were cloned into pmirGLO vectors (Promega, Madison, WI, USA). Afterwards, miR-128-3p mimic or mimic NC were co-transfected with the above vectors (SNHG1-WT, SNHG1-MUT, SNCA-WT, SNCA-MUT) into SH-SY5Y and SK-N-SH cells. A Dual Luciferase Reporter System (Promega) was adopted to detect the luciferase activity.

### 2.7. RNA Immunoprecipitation (RIP) Assay

To verify the relationship between miR-128-3p and SNCA, a Magna RIP RNA-Binding Protein Immunoprecipitation Kit (Millipore, Bedford, MA, USA) was used to conduct RIP analysis following the manufacturer’s manual. After collecting and lysing SH-SY5Y (2 × 10^7^ cells) and SK-N-SH cells (2 × 10^7^ cells), Argonaute2 (Ago2, Abcam)- or immunoglobulin G (Ig G, Abcam)-coated beads were applied to incubate cell lysates overnight at 4 °C. Then, miR-128-3p and SNCA from immunoprecipitated RNAs were detected using RT-qPCR analysis.

### 2.8. Data Analysis

All data were presented as means ± standard deviation (SD). GraphPad Prism 6 was used to analyze our data. Comparison of two groups were processed using Student’s *t*-test and comparisons of more than two groups were conducted using one-way analysis of variance (ANOVA). *p* < 0.05 was identified as a statistically significant difference. All experimental data were derived from three independent replicates. 

## 3. Results

### 3.1. RES Promoted Autophagy in MPP^+^-Induced Human Neuroblastoma Cells

**Figure 1:**
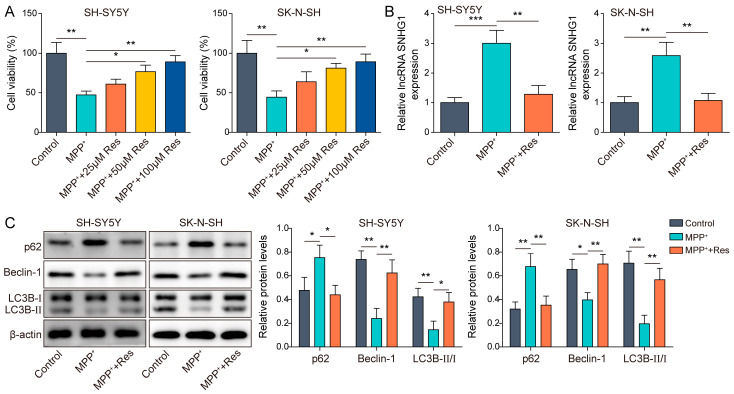
RES-promoted autophagy in MPP^+^-induced human neuroblastoma cells. (**A**) Cell viability was measured in SH-SY5Y and SK-N-SH cells with different concentrations of RES (25, 50 and 100 μM) pre-incubation and MPP^+^ treatment using CCK-8. SH-SY5Y and SK-N-SH cells were treated with 100 μM RES and followed by MPP^+^ induction. (**B**) qRT-PCR was used to assess SNHG1 expression. (**C**) Western blot was applied to detect p62, Beclin-1, LC3B-II and LC3B-I expression. All data were acquired from three replicate experiments. * *p* < 0.05, ** *p* < 0.01 and *** *p* < 0.001.

Primarily, SH-SY5Y and SK-N-SH cells were exposed with 1 mM MPP^+^ for 24 h to construct a PD model in vitro. To unearth the functions of RES in the cell model of PD, different concentrations of RES (25, 50 and 100 μM) were pre-treated with SH-SY5Y and SK-N-SH cells for 12 h and then the cells received 1 mM MPP^+^ induction for 24 h. Subsequently, we evaluated the functions of RES on cells through detecting some indicators. Firstly, we observed that, in two cell lines, MPP^+^ stimulation evidently inhibited cell viability, whereas RES with low-to-high concentration could gradually promote MPP^+^-suppressed cell viability (Figure 1A). Because 100 μM RES promoted cell viability to the maximum extent among 25, 50 and 100 μM RES, 100 μM RES was selected to run subsequent experiments. Of note, SNHG1 expression was evidently elevated in MPP^+^-induced human neuroblastoma cells. However, the elevated SNHG1 by MPP^+^ induction was compromised by 100 μM RES (Figure 1B). In addition, autophagy was reported to be closely related to the development of PD [8]. Therefore, we evaluated the cell autophagy of SH-SY5Y and SK-N-SH cells by detecting the expression levels of autophagy-associated proteins including p62, Beclin-1 and LC3B-II/LC3B-I. Encouragingly, western blot displayed that MPP^+^ induction dramatically upregulated p62 expression and decreased Beclin-1 expression and the ratio of LC3B-II/LC3B-I, indicating MPP^+^ treatment suppressed cell autophagy. However, RES pre-incubation inhibited these MPP^+^-generated alterations of autophagy-associated proteins (Figure 1C). These outcomes indicated RES reduced SNHG1 expression and promoted cell ability and cell autophagy in MPP^+^-induced SH-SY5Y and SK-N-SH cells.

### 3.2. SNHG1 Overexpression Reversed the Influences of RES on Autophagy in Human Neuroblastoma

**Figure 2:**
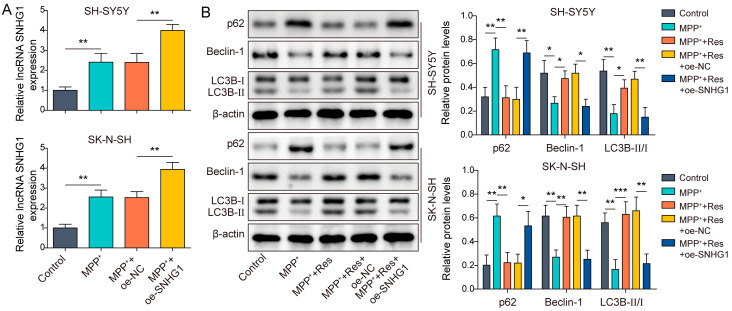
SNHG1 overexpression reversed the influences of RES on autophagy in human neuroblastoma cells. SH-SY5Y and SK-N-SH cells with/without oe-SNHG1 transfection were pre-incubated with 100 μM RES and then treated with MPP^+^ induction. (**A**) qRT-PCR was applied to evaluate the transfected efficiency of oe-SNHG1. (**B**) Western blot was employed to detect p62, Beclin-1, LC3B-II and LC3B-I expression. All data were acquired from three replicate experiments. * *p* < 0.05, ** *p* < 0.01 and *** *p* < 0.001.

Subsequently, we excavated the underlying molecular mechanism of RES on cell autophagy in cell model of PD. As presented in Figure 1B, we knew that SNHG1 expression was elevated by MPP^+^ treatment but suppressed by RES pre-incubation in SH-SY5Y and SK-N-SH cells upon MPP^+^ treatment, suggesting SNHG1 might be involved in RES-mediated influences in the MPP^+^-induced cell model of PD. To validate whether SNHG1 participates in RES-mediated influences in MPP^+^-induced SH-SY5Y and SK-N-SH cells, oe-SNHG1 was transfected into SH-SY5Y and SK-N-SH cells to elevate SNHG1 expression and then followed by MPP^+^ treatment for 24 h. SNHG1 expression was apparently elevated in cells with MPP^+^ treatment when compared to cells without MPP^+^ treatment (Figure 2A). As expected, SNHG1 overexpression further increased SNHG1 expression in MPP^+^-induced SH-SY5Y and SK-N-SH cells (Figure 2A). In addition, we measured how SNHG1 influences RES-mediated promotion of cell autophagy in MPP^+^-induced SH-SY5Y and SK-N-SH cells. Oe-SNHG1-transfected SH-SY5Y and SK-N-SH cells were subjected to MPP^+^ induction with/without RES pre-incubation. Our results revealed that MPP^+^ treatment promoted p62 expression and declined Beclin-1 expression and the ratio of LC3B-II/LC3B-I, while RES pre-incubation abolished these phenomena. However, SNHG1 overexpression inhibited RES-mediated elevation of Beclin-1 expression and the ratio of LC3B-II/LC3B-I as well as reduction of p62 expression (Figure 2B). Taken together, RES promoted cell autophagy in MPP^+^-induced SH-SY5Y and SK-N-SH cells via downregulating SNHG1 expression.

### 3.3. SNHG1 Knockdown Promoted Autophagy in MPP^+^-Induced Human Neuroblastoma Cells through Elevating miR-128-3p Expression

**Figure 3:**
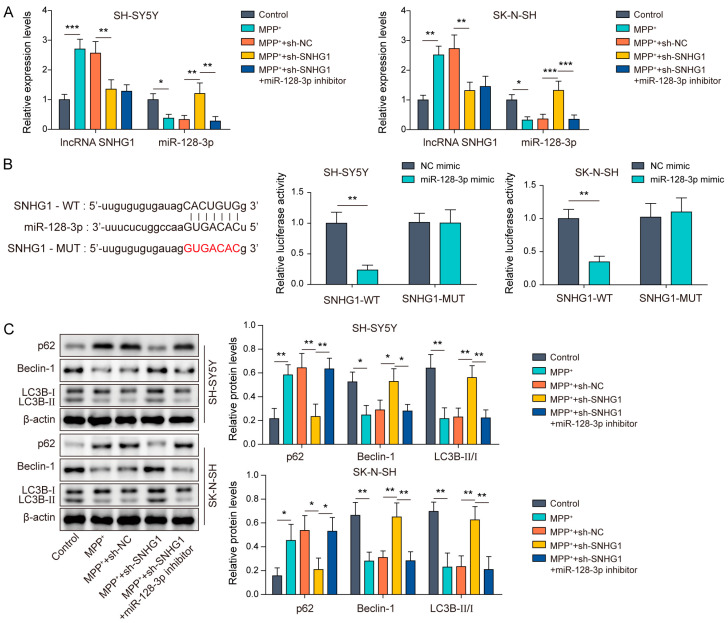
SNHG1 knockdown promoted autophagy in MPP^+^-induced human neuroblastoma cells through elevating miR-128-3p expression. SH-SY5Y and SK-N-SH cells were transfected with sh-SNHG1 or together with the miR-128-3p inhibitor upon MPP^+^ induction. (**A**) qRT-PCR was used to evaluate SNHG1 and miR-128-3p expression. (**B**) The binding site between SNHG1 and miR-128-3p was presented and a luciferase activity assay was used to validate the interaction between the two. (**C**) Western blot was performed to detect p62, Beclin-1, LC3B-II and LC3B-I expression. All data were acquired from three replicate experiments. * *p* < 0.05, ** *p* < 0.01 and *** *p* < 0.001.

We have known that SNHG1 was closely related with RES pre-incubation to PD, but the downstream molecules need to be further exploited. At present, several studies have indicated that miR-128-3p plays a protecting role in PD [17]. Intriguingly, we predicted that SNHG1 harbored binding sequences on miR-128-3p. Therefore, we speculated that SNHG1 might sponge miR-128-3p to engage in cell autophagy in MPP^+^-induced SH-SY5Y and SK-N-SH cells. To verify our conjecture, SH-SY5Y and SK-N-SH cells were transfected with sh-SNHG1 or in combination with an miR-128-3p inhibitor and then followed by MPP^+^ treatment. Here, we noticed that in MPP^+^-induced SH-SY5Y and SK-N-SH cells, SNHG1 expression was observably increased and miR-128-3p expression was notably reduced when compared to that in SH-SY5Y and SK-N-SH cells without MPP^+^ induction. However, SNHG1 knockdown effectively reversed the MPP^+^-induced elevation of SNHG1 expression and suppression of miR-128-3p expression (Figure 3A). It was noted that the miR-128-3p inhibitor abolished SNHG1 knockdown mediated elevation of miR-128-3p expression but had no impact on SNHG1 expression (Figure 3A). In addition, Figure 3B exhibited the binding site between SNHG1 and miR-128-3p through Starbase (Figure 3B). To validate the interaction between SNHG1 and miR-128-3p, a dual luciferase activity assay was conducted. The results revealed that miR-128-3p mimicked evidently inhibited luciferase activity in the SNHG1-WT group but failed to impact the luciferase activity in the SNHG1-MUT group (Figure 3B). As for the influences of autophagy, we found that SNHG1 knockdown could restore the MPP^+^-induced reduction of Beclin-1 expression and the ratio of LC3B-II/LC3B-I as well as the elevation of p62 expression, while these alterations were abolished by the miR-128-3p inhibitor (Figure 3C). In total, the miR-128-3p inhibitor eliminated the SNHG1 knockdown-mediated promotion of cell autophagy in MPP^+^-induced SH-SY5Y and SK-N-SH cells.

### 3.4. MiR-128-3p Targeted SNCA

**Figure 4:**
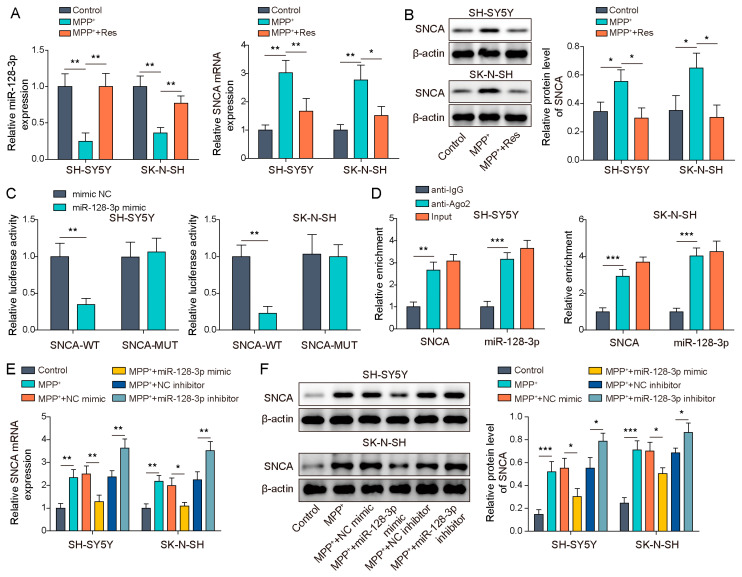
MiR-128-3p targeted SNCA. SH-SY5Y and SK-N-SH cells were pre-incubated with 100 μM RES and followed MPP^+^ induction. (**A**) qRT-PCR was performed to assess miR-128-3p and SNCA expression. (**B**) Western blot was performed to detect SNCA expression. (**C**,**D**) A dual luciferase activity assay and RIP were used to validate the interaction between miR-128-3p and SNCA expression. (**E**,**F**) qRT-PCR and western blot were performed to measure SNCA expression in SH-SY5Y and SK-N-SH cells with miR-128-3p mimics and inhibitor transfection upon MPP^+^ induction. All data were acquired from three replicate experiments. * *p* < 0.05, ** *p* < 0.01 and *** *p* < 0.001.

As is known to all, SNCA was a crucial molecule in regulating PD [21]. Furthermore, we predicted the binding site between miR-128-3p and SNCA. Firstly, SH-SY5Y and SK-N-SH cells were subjected to MPP^+^ induction with/without RES pre-incubation and miR-128-3p and SNCA expression was measured. We observed that miR-128-3p expression was evidently inhibited while SNCA expression was notably enhanced by MPP^+^ induction, while RES administration abolished these effects (Figure 4A,B). Subsequently, we verify the interaction between miR-128-3p and SNCA through conducting a dual luciferase activity assay and an RIP assay. The dual luciferase activity assay displayed that the miR-128-3p mimic attenuated luciferase activity in the SNCA-WT group rather than in the SNCA-MUT group (Figure 4C). Furthermore, the RIP assay exhibited that both miR-128-3p and SNCA were notably enriched by Ago2 (Figure 4D). These two results of the dual luciferase activity assay and the RIP assay suggested that miR-128-3p could interact with SNCA. In addition, in MPP^+^-induced SH-SY5Y and SK-N-SH cells, the miR-128-3p inhibitor could apparently elevate SNCA expression at mRNA and protein levels, but miR-128-3p overexpression led to the opposite result (Figure 4E,F). Altogether, miR-128-3p could negatively regulate SNCA expression through interacting with SNCA in MPP^+^-induced SH-SY5Y and SK-N-SH cells. 

### 3.5. SNHG1 Knockdown Prompted Cell Autophagy in PD through Reducing SNCA Expression

**Figure 5:**
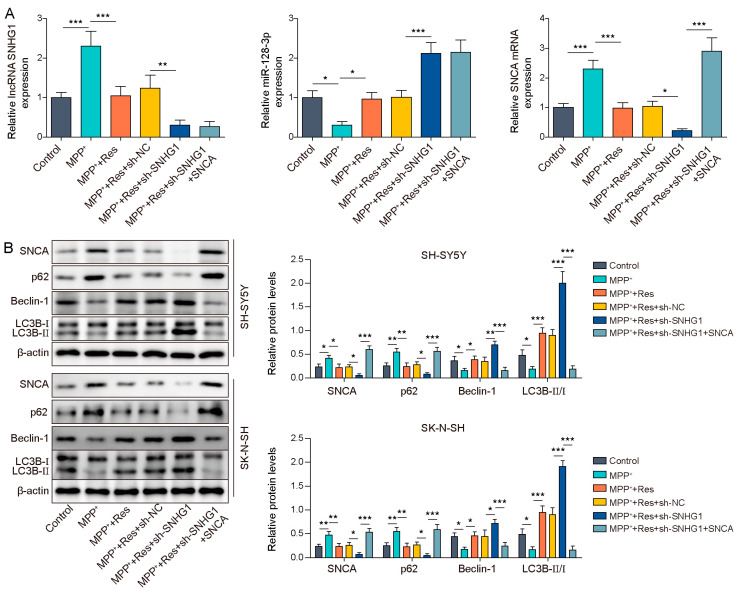
SNHG1 knockdown prompted cell autophagy in PD through reducing SNCA expression. SH-SY5Y and SK-N-SH cells were transfected with sh-SNHG1 and an SNCA overexpressing vector upon RES pre-incubation and MPP^+^ induction. (**A**) qRT-PCR was used to assess SNHG1, miR-128-3p and SNCA expression. (**B**) Western blot was applied to detect SNCA, p62, Beclin-1, LC3B-II and LC3B-I expression. All data were acquired from three replicate experiments. * *p* < 0.05, ** *p* < 0.01 and *** *p* < 0.001.

Here, we evaluated whether SNCA is implicated in sh-SNHG1-mediated promotion of cell autophagy in MPP^+^-induced SH-SY5Y and SK-N-SH cells upon RES pre-incubation. Primarily, sh-SNHG1 with or without an SNCA overexpressing vector were transfected into SH-SY5Y and SK-N-SH cells and then followed by RES pre-incubation and MPP^+^ induction. SNHG1 knockdown reinforced the RES-induced reduction of SNHG1 and SNCA expression and the elevation of miR-128-3p expression in MPP^+^-induced SH-SY5Y and SK-N-SH cells, whereas SNCA overexpression abolished SNHG1 knockdown-mediated reduction of SNCA expression but had no influences on SNHG1 and miR-128-3p expression (Figure 5A,B). With regard to autophagy-related proteins, MPP^+^-mediated the upregulation of p62 expression, as well as the reduction of the Beclin-1 level, and the ratio of LC3B-II/LC3B-I was reversed by RES pre-incubation. SNHG1 knockdown strengthened the RES-mediated effects of promoting autophagy, while SNCA overexpression reversed these effects caused by SNHG1 knockdown (Figure 5B). Taken together, SNCA overexpression suppressed the SNHG1 knockdown-mediated promotion of cell autophagy in MPP^+^-induced SH-SY5Y and SK-N-SH cells upon RES pre-incubation.

## 4. Discussion

PD gives rise to great inconvenience to normal life because of its symptoms, mainly including static tremor, bradykinesia, myotonia and postural gait disturbance. As reported previously, promoting autophagy probably enabled a decrease of α-synuclein aggregation to relieve PD [22]. RES has been reported as a promising drug for PD treatment due to its powerful functions [23]. In our study, we further proposed that RES possessed the capacities for activating cell autophagy to prevent PD via mediating the SNHG1/miR-128-3p/SNCA axis.

Significant investigations have reported that autophagy dysfunction was associated with the pathogenesis of many neurodegenerative diseases including PD [24]. Regarding the relationship between autophagy and PD progression, there were many reviews expounding the complicated correlation. For instance, Silvia Cerri summarized that promoting autophagy might be a promising approach for the disease-modifying treatment of PD [25]. In addition, rapamycin, an inducer of autophagy, was identified as a protector to improve cell injury caused by in MPTP/MPP+-induced models of PD [26]. Xue Zhang revealed that the inhibition of P2X4R expression resulted in promoted autophagy, which elevated the Beclin-1 level and the ratio of LC3B-II/LC3B-I and decreased p62 expression in 6-hydroxydopamine (6-OHDA)-induced PD to alleviate PD progression [27]. In this study, our findings also revealed that MPP+ induction inhibited cell viability and promoted autophagy in SH-SY5Y and SK-N-SH cells, which was proved by elevating p62 expression and declining the Beclin-1 level and the ratio of LC3B-II/LC3B-I.

Chinese herbal medicine is an important part of the source of Chinese medicine. In recent years, it has received a lot of scientific favor, showing its powerful role in fighting disease. Furthermore, various natural compounds, such as Trehalose, RES, and Curcumin, have been reported to be related by participating in autophagy in PD progression [25]. In this study, we concentrated on the functions and mechanism of RES on autophagy in PD progression. RES has been investigated by a lot of science researchers. There were numerous studies on RES, involving multiple disease systems, such as the cardiovascular system, the nervous system, the digestive system, etc. [28,29,30]. In PD, RES has the strong powers of oxidation resistance and the ability to regulate mitochondrial dysfunction [23], hinting that RES might be an available drug for PD treatment. Furthermore, previous studies have also reported the effects of RES on autophagy. For instance, RES attenuated the rotenone-induced apoptosis in SH-SY5Y cells through enhancing autophagic flux to mediate neuroprotection and, therefore, to improve PD [31]. Here, our findings revealed that in MPP^+^-induced human neuroblastoma cells, cell viability was evidently suppressed, but RES exhibited the capability of promoting cell ability in MPP^+^-induced human neuroblastoma cells. In addition, RES reversed the MPP^+^-induced elevation of p62 expression and the reduction of the Beclin-1 level and the ratio of LC3B-II/LC3B-I, suggesting RES promoted cell autophagy in MPP^+^-induced SH-SY5Y and SK-N-SH cells. Our results indicated that RES could prevent PD progression through promoting cell autophagy.

According to previous studies, RES was reported to regulate lncRNA MALAT1 to inhibit the apoptosis of neurons in PD [32]. Thereupon, we assumed RES might be regulated by lncRNA to mediate cell autophagy. SNHG1 was documented as a crucial molecule in PD. For example, SNHG1 silencing activated cell autophagy to mitigate PD through regulating the miR-221/222/p27/mTOR pathway [15]. In this work, MPP^+^ induction enhanced SNHG1 expression, while RES pre-incubation abolished this effect. Furthermore. SNHG1 overexpression reversed the promoting impacts of RES on cell autophagy. Therefore, our results initially revealed that RES played a preventive effect on PD through the decreasing SNHG1 expression.

Generally, lncRNAs could sponge miRNAs to participate in downstream genes’ expression, thereby affecting biological processes [33]. Javier Vargas-Medrano and co-workers discovered that the miR-128-3p level was elevated by FTY720-C2 (a derivative drug of FTY720 for PD treatment) treatment in dopaminergic MN9D cells using PCR arrays [34]. Zhang G et al. revealed that miR-128-3p repressed hippocampal neurons’ apoptosis to improve PD [17]. Through online website prediction and a dual luciferase activity assay, we found that SNHG1 bound to miR-128-3p. Moreover, we observed that miR-128-3p expression was inhibited by MPP^+^ induction in human neuroblastoma cells, while RES pre-incubation or SNHG1 knockdown elevated miR-128-3p expression. The miR-128-3p inhibitor reversed the SNHG1 knockdown-mediated promotion of cell autophagy in MPP^+^-induced human neuroblastoma cells. SNCA is considered to be an important marker in PD [35] and a high expression of SNCA accelerated development of PD [36]. Overexpressing SNCA inhibited the process of cell autophagy in PC12 cells [37]. In our results, MPP^+^ resulted in the upregulation of SNCA expression, which was downregulated by RES pre-incubation. In addition, SNCA was identified as a downstream gene of miR-128-3p and negatively regulated by miR-128-3p. As expected, SNCA overexpression abolished the SNHG1 knockdown-mediated promotion of cell autophagy in MPP^+^-induced human neuroblastoma cells upon RES pre-incubation. 

As previously documented, the crucial factors including SNHG1, miR-128-3p and SNCA were reported to be regulated by RES in PD [16,32,38]. However, there was no research about the relationship between the RES-regulated single molecule and autophagy. Furthermore, considering that miR-128-3p has a common binding site with SNHG1 and SNCA, it is unknown whether the SNHG1/miR-128-3p/SNCA axis is regulated by RES and further affects autophagy, thus participating in the progression of PD. In our study, our innovation points were mainly reflected in how RES played a preventive role in PD through the SNHG1/miR-128-3p/SNCA axis to promote cell autophagy. 

Although our study provided evidence that RES promoted autophagy in PD through regulating the SNHG1/miR-128-3p/SNCA axis, our study still has some limitations. Increasing evidence has demonstrated that cell apoptosis was closely related to PD development and MPP^+^ induction also resulted in cell apoptosis [39]. In our study, we focused on cell autophagy rather than cell apoptosis. Therefore, we did not detect cell apoptosis. In addition, according to published reports, RES is characterized by low bioavailability due to low solubility/permeability, light-induced isomerization, auto-oxidation and rapid metabolism [40]. In order to make RES more efficient for use, especially in PD, in which it needs to pass the blood–brain barrier, it is necessary to find a way to overcome the shortcomings of its low bioavailability. In the field of pharmacokinetic research, changing dosage forms is the most common way to improve drug availability. For dosage forms of RES, various novel agents such as nanotechnology-based drug carriers, microcapsules and exosome have been studied, which effectively promoted the bioavailability of RES [40,41,42]. 

## 5. Conclusions

We firstly put forward the mechanism that RES could mediate the SNHG1/miR-128-3p/SNCA axis to play a preventive effect on PD progression through promoting cell autophagy. Our results further provided substantially experimental evidence for a preventive approach to PD by RES. Moreover, SNHG1, miR-128-3p and SNCA were also indicated by our study to be regulated by RES, and it was further found that RES could promote autophagy by regulating the SNHG1/miR-128-3p/SNCA regulatory axis, thereby protecting human neuroblastoma cells and preventing PD. Our findings supported and suggested the notion that RES is a promising drug for the prevention of PD and crucial molecules including SNHG1, miR-128-3p and SNCA might be targets for the prevention of PD.

## Data Availability

Data is contained within the article.
